# Conductive Polymer-Based Hydrogels for Wearable Electrochemical Biosensors

**DOI:** 10.3390/gels10070459

**Published:** 2024-07-12

**Authors:** Dinakaran Thirumalai, Madhappan Santhamoorthy, Seong-Cheol Kim, Hyo-Ryoung Lim

**Affiliations:** 1 Digital Healthcare Research Center, Pukyong National University, Busan 48513, Republic of Korea; dinakaran@pknu.ac.kr; 2 School of Chemical Engineering, Yeungnam University, Gyeongsan 38544, Republic of Korea; santham83@yu.ac.kr (M.S.); sckim07@ynu.ac.kr (S.-C.K.); 3 Major of Human Bioconvergence, Division of Smart Healthcare, College of Information Technology and Convergence, Pukyong National University, Busan 48513, Republic of Korea

**Keywords:** hydrogels, nanocomposite, wearable, electrochemical sensors, conductive polymer

## Abstract

Hydrogels are gaining popularity for use in wearable electronics owing to their inherent biomimetic characteristics, flexible physicochemical properties, and excellent biocompatibility. Among various hydrogels, conductive polymer-based hydrogels (CP HGs) have emerged as excellent candidates for future wearable sensor designs. These hydrogels can attain desired properties through various tuning strategies extending from molecular design to microstructural configuration. However, significant challenges remain, such as the limited strain-sensing range, significant hysteresis of sensing signals, dehydration-induced functional failure, and surface/interfacial malfunction during manufacturing/processing. This review summarizes the recent developments in polymer-hydrogel-based wearable electrochemical biosensors over the past five years. Initially serving as carriers for biomolecules, polymer-hydrogel-based sensors have advanced to encompass a wider range of applications, including the development of non-enzymatic sensors facilitated by the integration of nanomaterials such as metals, metal oxides, and carbon-based materials. Beyond the numerous existing reports that primarily focus on biomolecule detection, we extend the scope to include the fabrication of nanocomposite conductive polymer hydrogels and explore their varied conductivity mechanisms in electrochemical sensing applications. This comprehensive evaluation is instrumental in determining the readiness of these polymer hydrogels for point-of-care translation and state-of-the-art applications in wearable electrochemical sensing technology.

## 1. Introduction

Wearable sensors (WSs) have gained considerable interest recently due to their promise for a number of applications. Electrochemical biosensors hold great promise as wearable chemical sensors for a variety of applications due to their high performance, inherent miniaturization, and low cost. A wide range of wearable electrochemical biosensors (WEBSs) have been developed for the non-invasive, real-time monitoring of electrolytes and metabolites in sweat, tears, saliva, and interstitial fluid as indicators of the health status of the wearer [1,2]. Using flexible devices such as wearable smart devices and biosensors, it is possible to detect and acquire effective signals. Through various assembly techniques, bioelectronics incorporates soft tissue materials with mechanical flexibility, biocompatibility, and high durability into electrical products to accomplish particular objectives [3]. WSs are developed using efficient transducers that can transform undetectable changes in physiological functions into electrical signals without influencing the users’ actions. To achieve this, sensor devices require soft and biocompatible substances that can adapt to the human body with viscoelastic properties [4,5].

**Figure 1:**
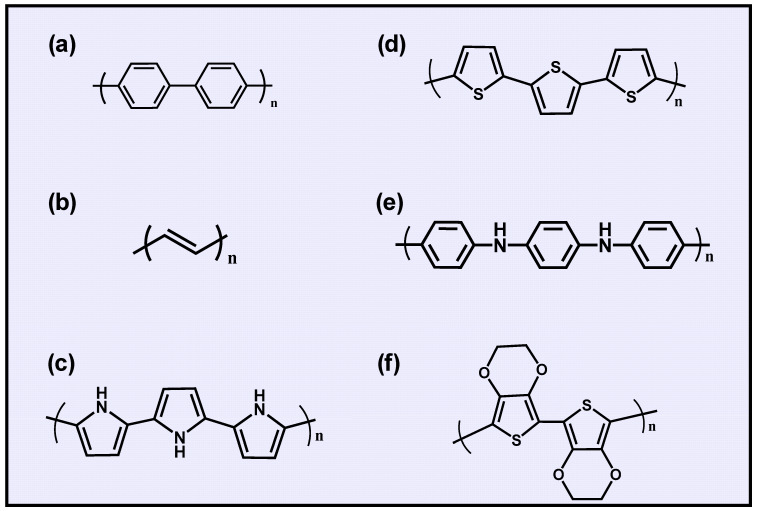
Chemical structures of conducting polymers: (**a**) poly(p-phenylene), (**b**) polyacetylene, (**c**) polypyrrole (PPy), (**d**) polythiophene (pTh), (**e**) polyaniline (PANI), and (**f**) polyethylene dioxythiophene (PEDOT).

Owing to advantages such as flexibility, easy synthesis, cost-effectiveness, and electrical and optical properties, conducting polymers (CPs) resemble metals and semiconductors. Furthermore, the physicochemical characteristics of CPs can be tunable by surface functionalization or doping approaches [6]. The different families of functional CPs are known as poly(p-phenylene), polyacetylene, polypyrrole (PPy), polythiophene (pTh), polyaniline (PANI), and polyethylene dioxythiophene (PEDOT) (Figure 1) [7,8].

These CPs allow lone-pair electrons to move and remain in their backbones. These lone-pair electrons move freely and provide a conductive conduit, which gives mobile charge carriers an electrical path and ultimately results in controlled conductivity. PPy, PANI, and PEDOT in particular are extensively utilized as electrode materials for stimulation and recording due to their enhanced biocompatibility and utility [9]. However, the application of CPs in WSs is limited due to their fragility and the complexity of handling them, as detailed in Table 1. 

A hydrogel (HG) is a soft material with a three-dimensional network structure formed by cross-linking hydrophilic macromolecules through covalent, hydrogen, or ligand bonds. Due to its unique porous structure and good flexibility, hydrogel presents excellent application prospects in flexible wearable electronics, batteries, and biosensors [10,11]. HGs are natural (for example, collagen, gelatin, alginate, cellulose, chitosan, and hyaluronic acid) or developed polymer chains that are linked to one another by cross-linkers to form an interconnected material with the macromolecular structure of a gel. They may contain up to 99% water or biological fluids, and they can swell to several times their dry weight [12]. HGs have porous features that give them good stretchability and elasticity [13]. They also have strong mechanical qualities and biocompatibility, which are traits shared with human skin tissues [14]. For this reason, HGs have gained attention lately as possible substrates for the creation of stretchy and flexible sensors. These three-dimensional (3D) highly hydrated porous networks are usually intended to store, release, or collect materials and can imitate the natural tissue microenvironment. HGs are widely used in the assembly of WSs, serving various functions including electrolyte storage, flexibility enhancement, and biological fluid sampling. Conductive polymer hydrogels (CP HGs), which combine the benefits of CPs and HGs, can be utilized as electrodes offering extensive specific surface area, electrical conductivity, and flexibility.

This review paper summarizes the recent developments in polymer-hydrogel-based WEBSs over the past five years. Polymer-hydrogel-based sensors, initially serving as carriers for biomolecules, have advanced to encompass a wider range of applications, including the development of non-enzymatic sensors, facilitated by the integration of nanomaterials such as metals, metal oxides, and carbon-based materials. One of the primary challenges in developing WEBSs is finding electrode materials with suitable mechanical properties. Traditional electrochemical biosensors are often rigid, heavy, and reliant on large electronic devices, making them unsuitable for wearable use. Transitioning from these hard, flat systems to soft, flexible, and small-scale devices enables seamless integration with the skin and the ability to endure repeated multi-directional mechanical stress. Flexibility, stretchability, self-healing capabilities, and transparency are crucial attributes for all WEBSs. CP HGs with superior electrical, mechanical, and swelling properties are being explored to address the above challenges in developing WEBSs. Going beyond the numerous existing reports that primarily focus on biomolecule detection, we extend the scope to include the fabrication of nanocomposite conductive polymer hydrogels. We also explore their varied conductivity mechanisms in electrochemical sensing applications and discuss their current limitations. This comprehensive evaluation is instrumental in determining the preparedness of these polymer hydrogels for point-of-care translation and state-of-the-art applications in wearable electrochemical sensing technology.

## 2. Conductive Polymer Hydrogels

Conductive polymer hydrogels (CP HGs), a new functional material, were first proposed by Guisep-pi-Elie et al. [15] and Wallace et al. [16] as a combination of hydrogels and conductive polymers. After more than a decade of development, they now exhibit high stability, electrical conductivity, and mechanical properties, making them highly useful in practical applications [17]. CP HGs are special hydrogels with high conductivity due to the incorporation of conductive elements into the hydrogel matrix [18,19]. They have been created using a range of conductive materials, including graphite materials, carbon nanotubes (CNTs), free ions, liquid metals, and conducting polymers [20]. Because of their excellent electrical and soft mechanical qualities, CPs are among the most promising materials for the creation of conductive hydrogels [21]. It has also been shown that conducting-polymer-based hydrogels offer a viable substrate for the development of stretchy and wearable sensors for real-world uses [22]. CP HGs are a novel form of soft functional material made of CPs with a porous structure and high water content.

### 2.1. Conductive Mechanism

Various research efforts have persistently focused on developing multifunctional CP HGs with satisfactory outcomes. These CP HGs are typically prepared using two approaches. One method increases electrical conductivity by using inherent metal matrix components, CPs, and carbon-based fillers. The other option is to increase ionic conductivity by incorporating salts or other charged elements into the hydrogel network [23]. By combining various conductive components and polymer networks, hydrogels may acquire a wide range of physical and chemical characteristics [10]. CP HGs offer strong electrical conductivity, mechanical characteristics, and biocompatibility, making them promising for use in wearable devices, biosensors, electronic skin, and other human-related applications [22].

**Figure 2:**
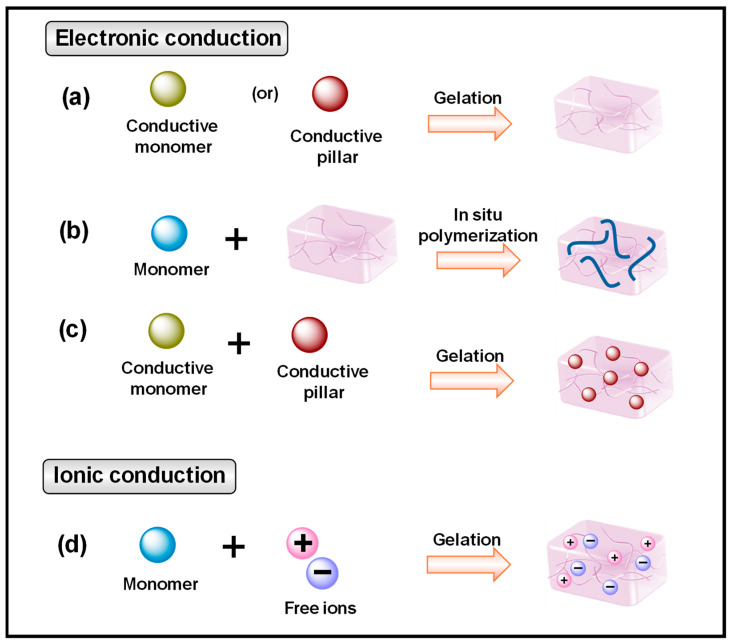
Conductive hydrogel fabrication and conductive mechanisms. (**a**) Direct gelation of conductive materials as cross-linking monomers, (**b**) suspending conductive materials within the hydrogel network, (**c**) in situ polymerization within a prepolymer hydrogel matrix, and (**d**) introduction of conductive ionic compounds.

The conducting efficiency of CP HGs is related to the concentration of doping or blending conducting materials. CP HGs show poor conductivity when the concentration of the conductive material is low in the CP HG matrix (Figure 2).

For example, flexible sensors can not only detect human mobility but also function as an intelligent health-monitoring system, tracking the body’s physiological data in real time. The key approach to preparing flexible biodevices involves combining the strong electrochemical conductivity of hydrogel characteristics with desirable physicochemical properties such as stiffness, viscoelasticity, swelling behavior, biodegradability, etc. CP HGs are mostly composed of PEDOT, PTh, PPy, and PANI, which combine to form a covalently or physically cross-linked hydrophilic network [24]. These types of CP HGs possess the benefits of hydrogel characteristics with distinct mechanical, chemical, and electrical characteristics, making them promising for use in wearable devices, biosensors, electronic skin, and other human-related applications. CP HGs undergo physicochemical characteristics when subjected to applied stimuli such as light, pressure, temperature, and chemical parameters. Advanced sensors require self-repairing capacities, high mechanical flexibility, and biocompatibility for next-generation wearable, portable, and implantable devices [25].

### 2.2. Fabrication of CP HGs

#### 2.2.1. General Synthesis Approach

The oxidative chemical or electrochemical polymerization method was applied to produce CPs in various forms, including powders, hydrogels, and films with different morphologies [22]. In the oxidative chemical polymerization process, an oxidant (ferric chloride) oxidizes the monomers to make radical cations, which then react to form dimers. These dimers are further oxidized and coupled to produce CPs [26]. With this approach, a huge quantity of CPs can be produced. Similarly, in the electrochemical polymerization approach, the applied electric field is utilized to oxidize the monomers [23]. Several preparation processes have been developed for 2D materials, including (i) top-down exfoliation and (ii) bottom-up growth [27]. Top-down exfoliation is effective when mechanical forces or ion/molecule intercalations are strong enough to break down interlayer connections. Mechanical exfoliation employs shear forces to break the van der Waals interaction between nanosheets. The bottom-up growth technique is particularly useful for producing ultrathin, high-quality nanocomposites with large lateral dimensions. Nanoscale materials are created through bottom-up processes from atomic or molecular sources, allowing two-dimensional materials to be produced efficiently and effectively.

Forming a porous 3D structure is an efficient way to prevent the restacking of 2D materials. The introduction of CPs into a 3D porous structure can result in high capacitance. Various strategies have been used to create three-dimensional composite aerogels and hydrogels with controlled porosity architectures [28]. 

#### 2.2.2. Copolymerization Techniques

In this copolymerization approach, conducting polymer monomers are polymerized onto nanostructured insulating hydrogel templates. Another method for making CP HGs involves copolymerizing CPs and insulating polymers to generate a composite hydrogel. In other words, the copolymerization procedure produces CP HGs by utilizing the distinct features of monomers, polymer chains, or components in the resulting hydrogel composite [29].

#### 2.2.3. Blending/Doping Method 

The doping or mixing of conducting components directly into the hydrogel matrix has been considered one of the straightforward approaches to fabricating CP HGs. The electronically conductive materials currently used primarily include metal-based materials, carbon-based materials, and CPs [21,30,31]. 

#### 2.2.4. Advanced Techniques for the Development of CP HGs

The development of fresh synthetic technologies has been one of the most important tactics for improving CP HGs’ practical applications. Preparing CP HGs with appropriate designs and meeting the needs of real-world applications remains a significant task. Three-dimensional printing technology has lately received a lot of attention and is regarded as an emerging adaptable additive manufacturing technique in the design of CP HGs due to its benefits of quick prototyping and customization [32]. Compared to previous procedures, 3D printing technology has several benefits, including a simple operating process, accurate structural control, and cost-effectiveness [33]. Three-dimensional printing methods are divided into five categories depending on their design and functioning mechanism: stereolithography (SL), digital light processing (DLP), bio-plotting, inkjet printing, and optical printing [34].

**Figure 3:**
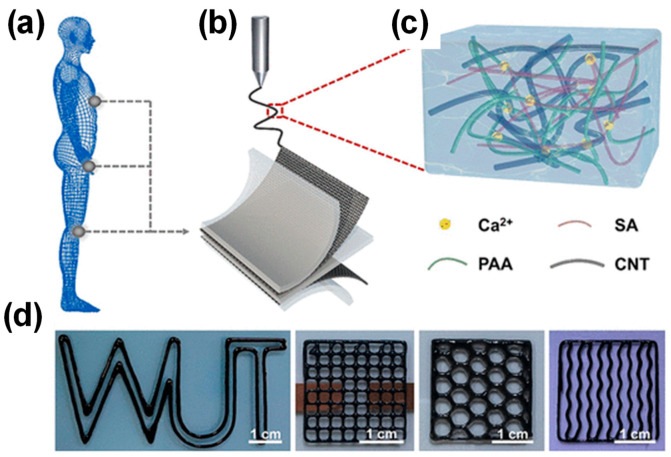
Fabrication of Ca-PAA-SA-CNT hydrogel-based strain sensor. (**a**) Human body movement sensing; (**b**) Ca-PAA-SA-CNT preparation by printing process; (**c**) prepared Ca-PAA-SA-CNTs HGs; (**d**) different 3D-printed patterns. Reprinted with permission from Ref. [37].

Nonetheless, SL has two drawbacks: long processing times and high costs. In contrast, DLP technology demonstrates short processing times, low costs, high accuracy, and multi-material processing [35]. Therefore, it has been used as an exceptional example of 3D printing technology in the production of CP HGs. For example, Caprioli et al. employed a commercial DLP printer to create a 3D CP HG with outstanding self-healing properties [36]. In another work, Wei et al. created a bioinspired CP HG utilizing DLP printing technology, integrating CNTs into a hydrogel matrix of polyacrylic acid (PAA) and sodium alginate (SA) (Figure 3). Due to 3D printing technology, this bioinspired CP HG demonstrated outstanding stretchability and diverse conductivities, making it easy to integrate into a strain sensor with simultaneous piezoresistive and piezocapacitive performance. In addition, CP HGs can be used as an advanced hydrogel in multifunctional skin-like smart wearable devices [37].

**Figure 4:**
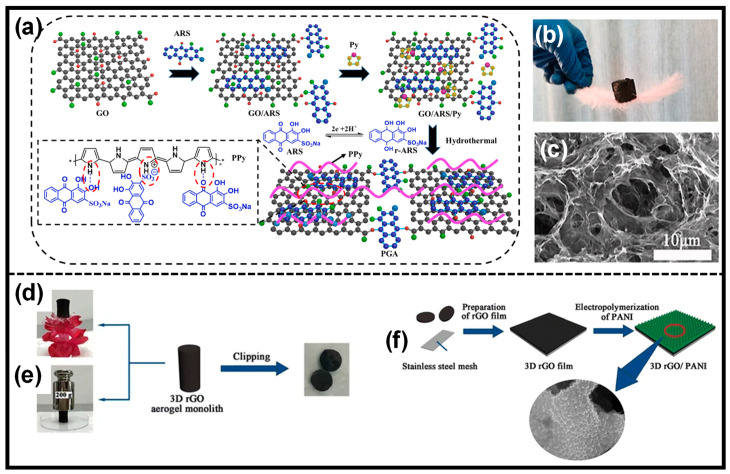
(**a**) Preparation method of rGO/PPy nanocomposite aerogel. The symbol (*) depicts the continuous attachment of the participating monomeric units. (**b**) Photo image of rGO/PPy composite aerogel standing on a feather. (**c**) SEM image of rGO/PPy composite aerogel. Reprinted with permission from Ref. [40] (Copyright 2022, Elsevier). (**d**,**e**) Mechanical properties of 3D rGO aerogel. (**f**) Preparation of PANI/rGO composite aerogel by electropolymerization. Reprinted with permission from Ref. [41] (Copyright 2017, Springer).

Because of its excellent gelation ability under high-pressure water vapor, GO is commonly used in the preparation of composite aerogels/hydrogels [38]. Ye et al. created a 3D hierarchical PPy/rGO aerogel with GO and PPy nanotubes as feedstock [39]. Instead of adding CPs to the GO dispersion, Han’s group used a hydrothermal technique to add a pyrrole monomer to the GO dispersion, resulting in a PPy/rGO hydrogel [40]. The PPy/rGO composite aerogel was produced following freeze-drying (Figure 4a). It was lightweight and could stand on top of a feather (Figure 4b). Figure 4c shows a typical 3D porous network topology for the composite aerogel. Conducting polymers can also be polymerized over rGO aerogels to create composite aerogels. For example, Yang et al. used electrochemical polymerization to make a PANI array/rGO composite aerogel (Figure 4d–f) [41]. MXene’s mechanical properties can be improved by employing a hydrogel matrix, such as polyvinyl alcohol (PVA). Zhang et al. developed an MXene-PVA hydrogel by repeatedly freezing and thawing it [42]. To make the composite hydrogel, PPy was chemically polymerized in a solution containing MXene and PVA hydrogel. The resultant PPy/MXene-PVA hydrogel exhibited an outstanding 10.3 MPa strength and could be stretched, knotted, and twisted.

There are various types of polymeric nanomaterials, including micelles, nanomaterials, core–shell particles, liposomes, and hyperbranched polymers. A large number of functional groups are found in the terminal region of branched polymer nanoparticles, resulting in higher reactivity. This property has attracted particular attention as it enhances hydrogel networks’ regularity and mechanical strength by cross-linking polymer chains with covalent or non-covalent bonds. The high stress-absorbing capacity of the generated NC HGs containing dendrimers makes them ideal for cartilage-tissue-engineering applications [43]. Liposomes have also sparked widespread attention due to their hydrophilic qualities, small size, biocompatibility, and potential for controlled drug delivery [44].

Metal matrix materials are mostly composed of metal nanowires or nanoparticles (e.g., Au, Cu, Ag, etc.). Metallic nanoparticles are commonly employed in the fabrication of CHs due to their high specific surface energy, electrical conductivity, and magnetic and catalytic characteristics [45]. For example, silver and gold nanoparticles are frequently used as conductive fillers in the preparation of conductive hydrogels due to their superior electron transport capacity as well as optical and catalytic capabilities. Traditional hydrogels are combined with metal-based particles and carbon nanoparticles during the polymerization of CPs [46]. The distinctive physical properties of metal and metal oxide nanoparticles, such as the conductivity of gold nanoparticles and the antibacterial characteristics of silver nanoparticles, have led to their broad application in creating nano-scaffold materials. These materials are extensively used in conductive scaffolds, switchable electronic devices, sensors, and actuators. Moreover, they serve as imaging agents and drug delivery systems in various biomedical applications [47]. Polyhydroxyethyl methacrylate (pHEMA) hydrogel, developed much later in 1960, appeared in the literature described by its typical hydrogel properties with high water affinity. The first-generation hydrogels are classified into three types. The first group includes polymers of alkene monomers exposed to radical-induced chain addition processes, primarily polyacrylamide (PAM) and polyhydroxyethyl methacrylate (pHEMA), which are still significant biomaterials despite being created more than 70 years ago. The second category includes covalently cross-linked hydrophilic polymers, such as polyvinyl alcohol (PVA) and polyethylene glycol (PEG), which are mostly utilized in tissue engineering. The third group includes cellulose-based hydrogels, which are mostly employed as drug dispersion matrices in drug delivery. The fundamental characteristic of third-generation hydrogels is “cross-linking”, which modifies the mechanical and degrading properties of hydrogels primarily via stereo conjugation, inclusion, metal–ligand coordination, and peptide chain synthesis. For example, one of the most common uses of stereo conjugation is the creation of injectable hydrogels by blocking two amphiphilic copolymers, poly(L-lactic acid) (PLLA) and poly(D-lactic acid). The hydrogels are classified according to their functionality, such as pH, temperature, and biomolecule responsiveness.

Lin et al. incorporated Ag nanoparticles onto hydrophilic cellulose nanocrystals (CNCs) to create hydrogels with high electrical conductivity [48]. Silver nanowires (Ag NWs) with high aspect ratios outperformed typical metal nanoparticles in terms of electrical conductivity. Jing et al. found that incorporating Ag NWs into sulfur gelatin (GE) hydrogels resulted in an effective electrical conduction channel and high conductivity [49]. Carbon-based materials, including activated carbon, carbon nanotubes (CNTs), porous carbon, carbon fibers, and graphene, offer excellent conductivity, stability, and low cost, making them ideal for creating conductive hydrogels [50,51]. Graphene and carbon nanotubes present a unique opportunity to create 3D conductive networks in polymer substrates. These materials allow electrons to travel across conjugated structures and possess excellent mechanical characteristics due to their diverse surface functions and high specific surface area [52]. They have been extensively studied in biosensing, flexible, and wearable bioelectronics. However, the hydrophobicity and low solubility of carbon nanotubes and graphene cause self-aggregation in aqueous conditions, making it difficult to interact with a hydrophilic hydrogel matrix. To address these issues, functional carbon-based compounds or hydrophilic substrates can be used to improve compatibility with hydrogels. For example, Gao et al. developed a hybrid hydrogel using carboxyl-functionalized MWCNTs. The electrostatic dispersion of MWCNTs in the hydrogel ensured good and stable conductivity [53]. Similarly, Han et al. successfully dispersed rGO with polydopamine (PDA) in a PAM hydrogel matrix, resulting in hydrogels with excellent mechanical and electrical characteristics [54].

Several other inorganic nanoparticles, including silica, calcium, phosphate, nano-hydroxyapatite, calcium phosphate, and bioactive glasses, have been used to improve the physical and biological properties of nanocomposite hydrogels (NC HGs). These mineral nanoparticles are required for normal tissue function in the human body [43,55]. Furthermore, it has been revealed that including nano-hydroxyapatite in the polymeric network increases the mechanical strength and physiological durability of nanocomposite matrices [56]. The addition of nano-hydroxyapatite reduces swelling in NC HGs by acting as a filler, and the interaction with the polymer network results in greater stiffness and less swelling than in pure hydrogels. The inclusion of polymers increases the interlayer gap and intercalated conditions. Clay mineral-loading NC HGs have garnered interest due to their unique compositions and properties [57]. Nano-sized platelets of clay minerals may be evenly distributed in the polymer matrix to significantly improve the mechanical and thermal characteristics of nanocomposites. Similar benefits are seen when clay minerals are added to polymeric hydrogels. Over the last decade, researchers have become more interested in the extraordinary toughness achieved by incorporating clay particles into nanocomposite hydrogels. Conducting nanoparticles can also alter the ordering and crystallization behavior of polymers, resulting in variations in optical anisotropy. Murata and Haraguchi discovered that when nanocomposite hydrogels with PNIPAM/LAPONITE^®^ XLG network architectures were distorted uniaxially, the optical anisotropy changed in novel ways [58]. Zhang et al. created a nanoclay cross-linked nanocomposite hydrogel with a bionic three-dimensional porous network structure composed of two-dimensional lamellar montmorillonite integrated into polyacrylamide via a cross-linking process, which improved the mechanical performance. The clay-based nanocomposite hydrogel had an expanded pore structure (11.7 µm) and a steady Young’s modulus (75.82 GPa) [59]. Self-assembly occurs not just in amphiphilic molecules, but also in inorganic material systems. Yang et al. created perovskite nanosheet-based poly(N-isopropylacrylamide) (PNIPAAm) hydrogels, with the nanosheets aligned to the gel surface at 300 nm intervals. The structural color gel demonstrated a linear and reversible mechanochromic response, detecting a weak stress of 1 kPa with a reaction time of less than 1 ms [60].

### 2.3. Properties of CP HGs

Hydrogels are cross-linked polymers with a three-dimensional network that can accommodate a large amount of water. The characteristics of hydrogels can be tuned by varying the synthesis processes. According to the structure and synthesis process of hydrogels, CP HGs are composed of inherently conductive materials and polymer networks. The conductivity of hydrogels varies depending on the conductive materials used. This section discussed several critical aspects of CP HGs needed for wearable sensor applications, including mechanical qualities, self-adhesiveness, self-healing, and biocompatibility.

#### 2.3.1. Electrical Conductivity

Due to their higher electronic and ionic conductivity, CP HGs are considered potential materials for electronic sensing devices. The conductivity of CP HGs ranges from 0.3 to 27 S/m, depending on the conductive polymer or doped substance [29]. Doped polymers have substantially greater conductivities (>10^4^ S cm^−1^) than undoped polymers (10^−6^–10^−10^ S cm^−1^). To enhance the electrical characteristics of CP HGs, highly conductive elements including carbon-based materials, conjugated polymers, liquid metals, and ionic or electronic conductive fillers are frequently incorporated into the polymer hydrogel matrix [61]. CP HGs incorporating conductive nanomaterials such as carbon- and metal-based compounds and MXene exhibit both ionic and electronic conductivity, with conductivity 1 to 5 orders of magnitude greater [62]. 

Due to their high conductivity, CP HGs have a large strain range and outstanding stability for strain sensor devices, extending their practical applicability in wearable sensor devices. He and colleagues created a unique electroconductive hydrogel using polymeric nanofibers. The prepared electroconductive hydrogel contained PPy, aramid nanofibers, and PVA. The suggested electroconductive hydrogel demonstrated significant conductivity (80 S·cm), structural resilience, mechanical strength (9.4 MPa), and fine stretchability (36%) without losing water content, making these hydrogels suitable for electrophysiological applications [63]. Ciarleglio and colleagues created a hybrid electro-conductive and thermosensitive hydrogel utilizing PNIPAM polymer and multi-walled carbon nanotubes in a two-step polymerization procedure. The results showed that the hydrogel had improved sensitivity and outstanding electroconductivity [64], suggesting that electroconductive hydrogels are viable candidates for wearing bioelectronics. 

Wang et al. developed a stereolithography-based method for creating conducting polymer hydrogels with complex lattice architectures. They showed that the created CP HGs had improved mechanical and electrical characteristics under strain, which were programmable via architecture and separated from the bulk qualities infused by their chemical compositions, eliminating the limitations associated with previous methods [32].

#### 2.3.2. Mechanical Properties

**Figure 5:**
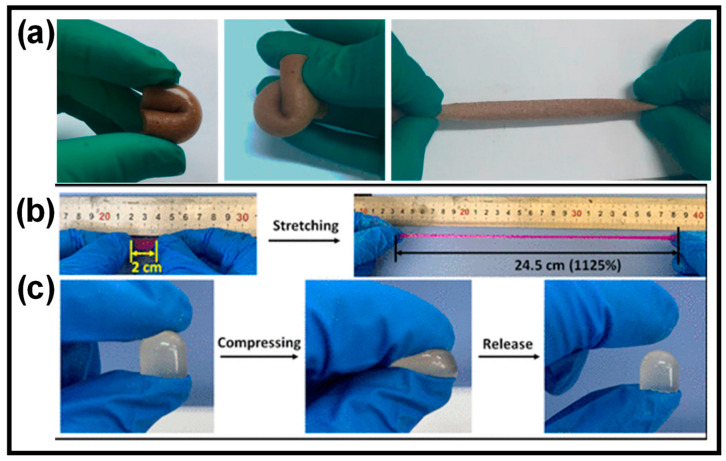
(**a**) Porous CP HGs with GO and PVA show excellent mechanical properties: bend, curl, and stretch. (**b**,**c**) PAA-OSA-AG HG mechanical properties. Reprinted with permission of Refs. [65,70].

Compared to typical hydrogel materials, CP HGs demonstrate superior mechanical and tunable characteristics. The porosity of CP HGs significantly impacts their mechanical characteristics as it affects free volume content, size, and surface properties [24]. Hollow sphere and porous nanostructured CP HGs have excellent mechanical qualities. Zhang et al. created a highly flexible porous polyvinyl alcohol (PVA) hydrogel with high tensile and compressive stresses of 400% and 80%, respectively (Figure 5a) [65]. The extraordinary mechanical properties of the porous CP HGs could be attributed to their novel binary network architecture or physically cross-linked networks. Additionally, the porous CP HGs had excellent mechanical properties due to hydrogen bonding interactions and crystallization sites [66].

Elasticity is essential for increasing the flexibility of the cross-linked network and facilitating the mobility of integrated medicinal moieties. To maintain the balance of mechanical strength and elasticity, hydrogels require an ideal degree of cross-linking. For example, PAAm–latex particle hydrogels had a low tensile strength, toughness, and elastic modulus of 19 kPa, 0.41 MJ m^−3^, and 25 kPa, respectively [67].

Good mechanical strength is considered a significant parameter for CP HG-based strain sensors to retain their integrity and performance under varying stress environments. An efficient hydrogel should possess sufficient mechanical strength, which includes good adhesiveness, tensile strength, ductility, toughness, and hardness. In this context, the double network (DN) approach is applied to develop CP HGs with enhanced mechanical strengths. Due to their distinct structural networks, DN-based CP HGs exhibit outstanding mechanical strength and elasticity. In the DN approach, two different polymers with physically opposing properties create a linked network [68]. The first network is rigid, while the second is ductile. As a result, DN-CP HGs consist of two interpenetrating cross-linked networks that are bonded into a soft material matrix, and their mechanical characteristics can be easily altered by varying the compositions of each network. DN-CP HGs can be robust or soft, with high failure tensile stress and strain, hardness, and toughness [69]. For example, Zhao et al. developed multifunctional ionic DN-CP HGs with good stretchability by mixing oxide sodium alginate (OSA), aminated gelatin (AG), and acrylic acid (AA) (Figure 5b,c) [70]. Physical metal coordination and dynamic Schiff base bonds enhanced the stretchability of these CP HGs.

Li et al. straightforwardly created a graphene-based flexible substrate employing PDMS and graphene oxide. The substrate demonstrated high sensitivity and a rapid reaction time (43 ms). The designed device effectively measured conveyor belt strain under high pressure (1.2–1.4 MPa) and high temperature (150 °C), illustrating the viability of utilizing flexible strain sensors in the field of flexible sensors [71]. Fu and colleagues developed a rationally designed strain sensor made from a gelatin and cellulose-derived hydrogel, featuring enhanced mechanical robustness, cryogenic durability, and flexibility through the synergistic reinforcement of powerful hydrogen bonds, imine bonds, and sodium bonds. They showed that synergetic triple dynamic bonds dominated by strong hydrogen bonds and assisted by imine and sodium bonds with higher strength can dissipate more mechanical energy. This provided the hydrogel with a 38-fold increase in tensile strength (6.4 MPa) and a 39-fold increase in toughness (2.9 MPa). They also proved that this hydrogel may function as a strong and biodegradable strain sensor with exceptional flexibility, a wide detection range, high sensitivity, and outstanding sensing stability [72].

#### 2.3.3. Self-Healing Property

Sensor electrical equipment may lose or degrade performance due to mechanical damage or repeated deformation, resulting in a reduced service life. As a result, one of the most important characteristics of CP HG-based sensor systems is their capacity to heal themselves. CP HGs with a high self-healing capacity can recover their structure, mechanical characteristics, and functionality following damage [73]. The self-healing capacity of CP HGs is attributed to two types of bonding: non-covalent bonds and dynamic covalent bonds. Non-covalent connections include host–guest interactions, metal coordination, hydrogen bonding, and hydrophobic interactions [74].

Ren et al. fabricated self-healing CP HGs based on aminated gelatin, dialdehyde alginate, and PPy. PPy was polymerized via an oxidizing agent, ammonium persulfate (APS), in a mixture of gelatin and alginate derivatives at −20 °C. Subsequently, the solution temperature was raised to allow for the formation of imine bonds and to create CP HGs. The mechanical strength of the obtained hydrogel reached 0.5 MPa, and the electrical conductivity, recalculated from the resistance, reached 1.4 × 10^−5^ S/cm [75].

Yang et al. investigated the combination of multi-walled carbon nanotubes (MWCNTs) with polyacrylamide hydrogel, utilizing cellulose nanofibers (CNF) as a dispersant. The self-healing character was provided by the hydrophobic interaction between the CNF and polyacrylamide chains. MWCNTs provided good electrical conductivity (8.5 × 10^−3^ S/m) while the CNF supported the mechanical strength, reaching 0.24 MPa. The real-time self-healing was achieved in 10 min, allowing the material to recover its original shape [76].

Liu et al. fabricated a self-healing hydrogel based on a regenerated silk fibroin (SF) substrate. They introduced beta-cyclodextrin (β-CD) molecules to the SF backbone through a reaction with monoaldehyde β-CD and amino groups present in the SF structure. The self-healing mechanism relies on the host–guest interactions between β-cyclodextrin and the aromatic groups of the amino acid side chains of SF, such as tyrosine, tryptophan, phenylalanine, and histidine. To enhance these host–guest interactions, ethynylbenzene groups were also attached to the SF backbone using an azo bridge [77].

**Figure 6:**
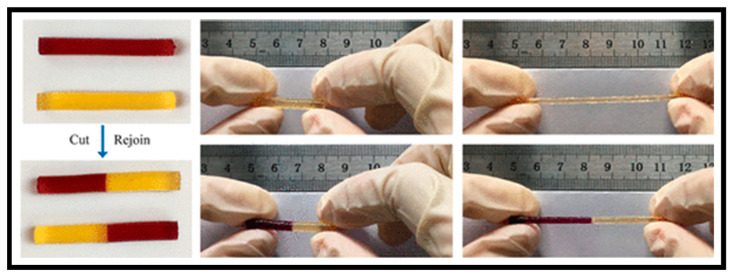
Self-healing of cut HGs after rejoining, and the stretching behavior of the healed HGs. Reprinted with permission of Ref. [79].

Dynamic covalent bonds, unlike covalent bonds, are reversible and stable [78,79]. To construct self-healing CP HGs, dynamic covalent couplings such as imine bonds [80], acyl hydrazone bonds [81], phenylboronate esters [82], disulfide bonds [83], and Diels–Alder reactions [84] are often employed. Because of its simplicity and lack of catalyst support, the imine bond produced by the Schiff base reaction has been widely employed for dynamic covalent bonding [85]. Furthermore, the Schiff base reaction can be carried out quickly and efficiently under neutral conditions and without external inputs. For example, Lei et al. recently developed a biocompatible gelatin-based self-healing CP HG containing an imine bond generated by the Schiff base reaction between gelatin and cellulose without additional cross-linking agents (Figure 6) [79]. Because gelatin has many amino groups, the Schiff base cross-linkages can be induced to form a hydrogel by dynamic imine connections between gelatin and cellulose. This results in hydrogels that are extremely flexible and soft when squeezed and bent. Furthermore, the hydrogel can self-heal after 30 min of cutting and reconnecting, and the restored hydrogel can withstand high stress. Healing proficiency can reach 90% within 60 min after treatment, demonstrating the exceptional self-healing abilities of CP HGs.

#### 2.3.4. Adhesion Property

In addition to their superior mechanical properties, CP HGs exhibit adequate adhesive ability, which is essential for sensitive sensing capabilities. Strong and stable adhesion to substrates and devices can prevent interfacial failures and functionality loss in CP HGs, significantly increasing the reliability and efficacy of sensor devices [86]. CP HGs can adhere to various substances, including glass, metal, ceramics, polymers, and human skin [87]. To achieve high adhesive properties, two key elements are necessary: superior mechanical properties and strong interfacial bonding between the hydrogel and the substrate material. As a result, stealth or inert materials are widely used to make self-adhesive CP HGs, as they can prevent biomacromolecule attachment while retaining hydrogel properties [88]. For example, methacrylic acid, which contains vinyl and carboxyl groups, has been used in CP HGs to increase hydrogel adhesion to various substrate surfaces [89].

CP HGs with good adhesion and biocompatibility are feasible candidates for strain sensors in human health monitoring. CP HGs bind to hydrogels through various modes of interaction, including hydrogen bonding, hydrophobic action, metal complexation, and π-π stacking with appropriate amine or carboxylic acid groups [90]. These physical interactions cause CP HGs to have remarkably repeatable adherence. Ma and coworkers demonstrated novel CP HGs with strong adherence that employed bases from adherent DNA [91]. Hydrogen bonds, chemical cross-linking, and an ionic cross-linking dynamic network constructed the self-healing hydrogel.

Tannic acid, a type of polyphenol compound, contains numerous functional groups, including carbonyl, phenolic, and hydroxyl groups, which can interact with various organic and inorganic substrates via hydrogen bonding, electrostatic interaction, coordination bonds, and hydrophobic interactions. Tannic acid is frequently used to create self-adhesive conductive hydrogels. For example, Fan et al. immersed the generated PVA aerogels in tannic acid to create a dual cross-linked hydrogel with outstanding self-adhesive ability (60–80 kPa) [92].

Wang et al. described an ultra-stretchable, highly sticky, and self-healing ionic hydrogel that fully utilized tannic-acid-enabled dynamic interactions. These interactions not only improved the hydrogel’s bulk performance by providing exceptional stretchability (7300% for fracture strain) and outstanding self-healing capabilities, but they also influenced the hydrogel’s interfacial characteristics, resulting in strong adhesion (particularly 50 kPa on porcine skin). Tannic acid and calcium ions (Ca^2+^) form hydrogen bonds and coordination bonds, significantly increasing tensile stress (from 6.23 kPa to 24.51 kPa), elongation (from 593% to 6342%), and mechanical strength (54 kPa) [93].

#### 2.3.5. Biocompatibility

Wearable or implantable devices have close contact with the human body and are regarded as safe for human health [94]. CP HGs are an excellent alternative for biocompatible materials due to their unique hydrated environment and configurable physicochemical properties. They have been widely used in several biological devices, including biosensors, tissue engineering, controlled drug administration, and cell cultures. CP HGs, which have an organic composition similar to that of biological tissues’ extracellular matrix, have emerged as a promising candidate for next-generation bioelectronic interfaces as a bridge between biology and technology.

Combining CP HGs with natural polymer materials has garnered significant interest as a strategy for creating biopolymeric biomaterials. Chitin, alginate, and gelatin have all been investigated as biocompatible and biodegradable functional materials for the production of biocompatible CP HGs [95]. Natural polymers can increase cell adhesion and produce physical cross-links, hence improving the mechanical properties of CP HGs. Gan et al. developed a biocompatible hydrogel containing PPy, CS, and PAM [96]. This composite hydrogel exhibits good drug delivery, cell adhesion, and wound-healing properties. When compared to the control group, it can accelerate wound healing and increase tissue integrity. CP cryogels with microporous structures, a specialized type of CP HG formed at subzero temperatures, have become crucial tools in biomedical research [97]. They have outstanding properties such as macroporosity, tissue sample flexibility, and biocompatibility, which are valuable in microbiological applications. Humpolícek et al. discovered that PANI cryogels are more biocompatible than normal polyaniline due to lower impurity levels [98].

## 3. Applications of Wearable Electrochemical Biosensors (WEBSs)

Non-invasive monitoring of chemical indicators in WEBSs is a rapidly developing digital health technology. Wearable sensors have attracted much scientific interest over the last several years. Early research efforts aimed at developing wearable sensor devices for drug misuse were mostly restricted to detecting drug-related alterations. Examples include a wristband that monitors drug misuse by measuring changes in pulse rate, skin temperature, and conductance, and a wearable sensor that uses drug-induced low heart rate to trigger antidote administration to combat drug overdose [99]. Although wearable sensors have gained popularity, they cannot directly detect drugs and instead rely on monitoring bodily indicators, which can be influenced by other factors such as stress and anxiety. To address these issues, significant research efforts have recently focused on developing wearable devices capable of direct and continuous monitoring of medications. 

WSs provide real-time data, allowing individuals to track their ideal health conditions throughout their daily lives. Based on chemical information transduction, non-invasive chemical and biochemical sensors are still in their infancy compared to physical wearable sensors for signal monitoring. The scarcity of chemical sensors has slowed their development towards continuous personal health monitoring. Several critical issues, including achieving sensor response with low analyte concentration, a tiny sample volume of biofluid, and sensor biocompatibility, have not been satisfactorily solved [1].

Non-invasive WEBSs can detect target analytes in tears, sweat, and saliva, similar to their in vitro counterparts. Much research has gone into creating an effective biosensor for monitoring these biofluids. WEBS devices have garnered interest due to their advantages, including quick sensing capabilities, affordability, and selectivity towards key electroactive pharmaceuticals [100]. 

Hydrogel-based WEBSs have emerged as a promising technology in this field, offering several benefits such as soft structures, biocompatibility, superior biomolecule immobilization capabilities, and high sensitivity [101].

### 3.1. WEBSs for Glucose Monitoring

The concentration of glucose in the blood is an important measure of a patient’s health and condition. Because of the significant growth in diabetes rates, multiple researchers are working to create new glucose testing techniques to accurately assess the level of glucose in the blood. Despite the need for the direct monitoring of blood glucose levels, many diabetes patients are hesitant to employ invasive needle-based blood collection. As a result, non-invasive approaches are considered a hot research topic. The blood glucose level in sweat corresponds to the actual blood glucose level; hence, the real-time determination of the glucose level in blood using a wearable sensor can represent the patient’s health state. However, assessing glucose levels in sweat is difficult due to the low glucose concentration. As a result, very sensitive sensing technologies must be developed, particularly for detecting glucose in sweat even in the presence of contaminated skin residues [102].

**Figure 7:**
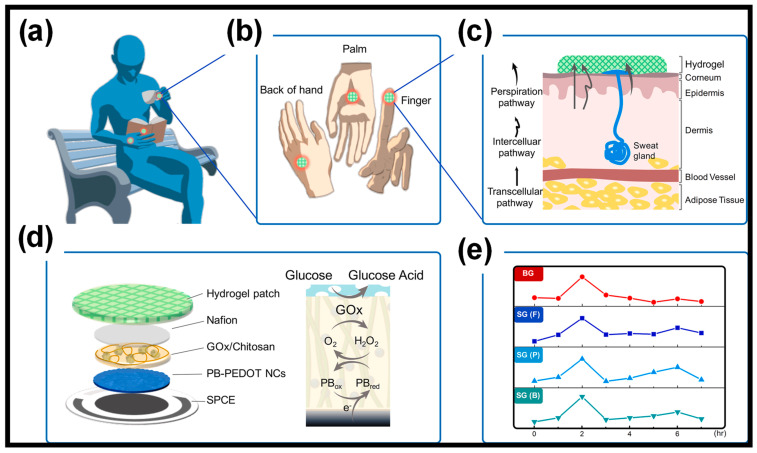
(**a**) The hydrogel patch is versatile, allowing for sweat sampling at various locations during periods of rest. (**b**) It can be placed on the finger, palm, or back of the hand for efficient sweat collection. (**c**) It utilizes preferential glucose pathways to sample sweat naturally through the hydrogel patches. (**d**) The sweat glucose sensor features a multi-layered enzymatic PB-PEDOT NC electrode and operates with a GOx mechanism alongside a PB probe. (**e**) This device is capable of monitoring sweat glucose levels without the need for high-intensity activity or external stimuli, at various sites including the finger (F), palm (P), and back of the hand (B). Reprinted with permission from Ref. [110] (Copyright 2022, Elsevier).

Over the past decade, several wearable prototypes have been developed for sweat glucose monitoring [103,104,105,106,107,108,109]. One such hydrogel-patch-based WEBS reported (Figure 7) a non-invasive sweat glucose sensor for the rapid sampling of natural perspiration without the external stimulation of sweating [110]. They optimized the surface of the Prussian Blue (PB)-doped poly(3,4-ethylenedioxythiophene) nanocomposite electrode, greatly improving the non-invasive sweat glucose measurement with excellent stability and electrocatalytic activity. Aycan et al. [111] developed a hyaluronic-acid-based hydrogel sensor that demonstrated notable electrochemical responsiveness to glucose molecules, characterized by a limit of detection (LOD) of 0.3 µM, as well as high sensitivity (421.42 µAmM^−1^cm^−2^), and selectivity amidst various interfering substances like uric acid, ascorbic acid, and sorbitol, along with outstanding long-term stability. The incorporation of reduced graphene oxide (rGO) and PANI into the hydrogel structure not only conferred efficient electrical conductivity but also enhanced mechanical properties, such as compressive strength and elastic modulus, which are essential for sensor applications.

Liang et al. [112] developed a self-healing hydrogel using quaternized chitosan and oxidized dextran, which was modified with cerium oxide and manganese oxide hollow nanospheres. The nanospheres were covalently immobilized into the hydrogel using 1-Ethyl-3-(3-dimethyl aminopropyl) carbodiimide/N-Hydroxy succinimide (EDC/NHS) coupling, preventing leaching and sustaining sensing performance. The nanospheres were biocompatible, inexpensive, and beneficial for glucose oxidase (GOD) immobilization. The hydrogel enriched glucose, enhancing glucose oxidase contact, sensitivity, and response rate. A flexible polyethylene terephthalate film and a gold-based three-electrode screen-printing chip were coated with nanosphere-encapsulated hydrogel, and glucose was successfully tested in physiologically relevant ranges. The excellent performance was attributed to the large specific surface area of the hollow nanospheres and the hydrophilic environment of the polymeric network. This flexible system has potential applications as a continuous glucose-monitoring system. Flexible GOD/PB/rGO/SF (Silk Fibroin) composite membrane [113], a conductive hydrogel with Pt/MXene-based microfluidic patches [106], a self-healable glucose adaptive hydrogel-based triboelectric-induced sensor [114], a PEDOT:PSS hydrogel with PB and GOD [115], and Ni-Co MOF/CNTs/MWCNTs/PDMS [116] for glucose-monitoring sensors were also reported recently (Table 2).

### 3.2. WEBSs for Lactate Detection

Lactate or lactic acid is mostly a byproduct of the anaerobic digestion of sugar. Lactic acidosis is produced by the increase in lactic acid in the human body during intense activity, resulting in metabolic acidosis. Lactic acidosis can also develop in newborns who have diarrhea, hypoxia, and bleeding. The clinical causes of lactic acidosis are classified as follows: shock, diabetes, infection, liver illness, and hypoxia. The key symptoms of lactic acidosis include low blood pressure, deep and fast breathing, vomiting, and, in extreme instances, unconsciousness [117,118]. 

**Figure 8:**
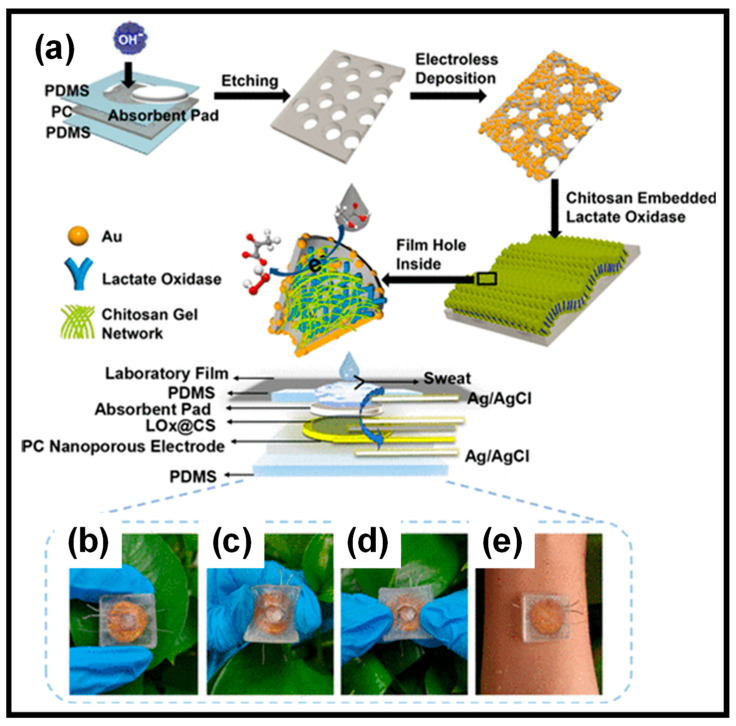
(**a**) Preparation of the LOx@CS PC sensor. (**b**–**e**) Photographs of the sensors prepared for measuring lactate in sweat. Reprinted with permission from Ref. [120] (Copyright 2024, American Chemical Society).

Commonly, lactase oxidase (LOx) is the main enzyme used in the wearable sweat lactate sensors that were reported [119]. Wu et al. [120] described a WEBS that featured gold nanoelectrode arrays on a nanoporous polycarbonate (PC) membrane (Figure 8). This sensor, which encapsulates LOx within a chitosan (CS) hydrogel, can simultaneously detect body temperature and sweat lactate levels. The nanoporous electrode significantly reduces resistance and offers nanoconfined spaces that enhance the LOx catalytic reaction. It also limits substrate concentration on the surface, minimizing substrate inhibition, which leads to a wider detection range and greater selectivity.

Yao et al. [121] developed a 3D lactate electrochemical sensor using a conductive scaffold composed of polydimethylsiloxane (PDMS) layered with PEDOT-coated CNT, PB, and LOx. This structure was integrated into a collagen hydrogel containing C6 glioma cells. The LOx catalyzed the oxidation of lactate to pyruvate and hydrogen peroxide (H_2_O_2_). The sensors, which included PB alongside CNT, showed sustained electrochemical stability in detecting H_2_O_2_. In addition, a conducting NC HG was based on a mixture of alginate and PEDOT, which was loaded with gold nanoparticles and LOx [122], CNCs modified by polydopamine, and gold nanoparticles into the poly(vinyl alcohol) network, which provided enhanced conductivity and mechanical strength, and immobilized LOx [123] for lactate detection.

Saha et al. developed a wireless electrochemical technique for the continuous measurement of sweat lactate, employing a functionalized hydrogel for osmotic sweat extraction and a paper-based microfluidic device for easy sweat transport. An electrochemical lactate sensor, screen-printed and connected to a potentiostat system, was integrated with the microfluidic device, enabling the immediate detection of trace sweat lactate levels with minimal power usage, all monitored by the potentiostat. This wearable sensor monitors sweat lactate levels across various exercise intensities with fluctuating lactate concentrations [124]. The analytical performance of recently reported WEBSs for lactate detection is summarized in Table 3.

### 3.3. WEBSs for Other Biomarkers

The electrochemical sensor based on the tannic acid–Ag–carbon nanotube–polyaniline (TA-Ag-CNT-PANI) composite hydrogel was designed for the on-body detection of pH and tyrosine (Tyr), a disease marker associated with multiple disorders, such as tyrosinemia and bulimia nervosa. The wearable sweat sensor can not only monitor pH and Tyr levels in sweat simultaneously, but also further calibrate Tyr detection results with the measured pH value to eliminate the effect of Tyr response variance at different pH levels and enhance the accuracy of the sensor [125].

**Figure 9:**
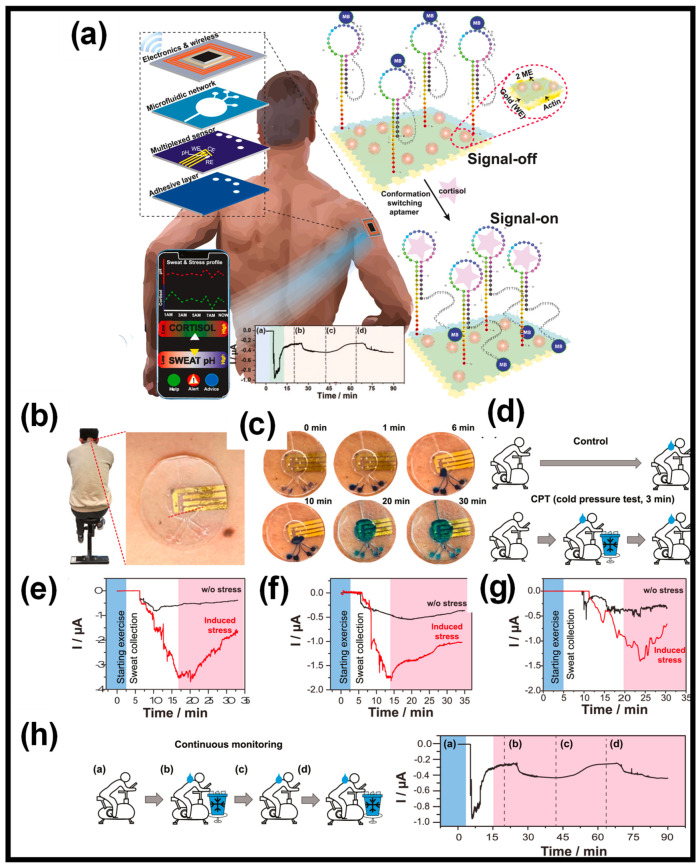
(**a**) Depiction of a wearable sweat patch designed for cortisol level monitoring. This device integrates a microfluidic system with an electrochemical sensor that captures, processes, and instantaneously detects cortisol levels. It employs a conformation-switching aptamer featuring a pseudoknot structure. The measured cortisol concentrations and sweat pH levels can be wirelessly transmitted for visualization and analysis to an adjacent smart device, and measurement of on-body signal regeneration (a–d). (**b**) Application of the sweat patch on the lower neck of participants during trials. (**c**) Photographs of the microfluidic reservoir filling with sweat during physical activity, with blue dye added to each inlet for visual confirmation. (**d**) Test protocol, including control and stress induction phases. (**e**–**g**) Continuous monitoring of sweat from three distinct individuals. (**h**) Long-term continuous monitoring, (h-a) Initial period of exercise, (h-b) Rapid increase after sweat fills the reservoir, (h-c) Subsequent restoration to a normal level in 7–8 minutes, (h-d) Stress was induced by a cold pressure test, corresponding measurement of on-body signal regeneration (a–d). Reprinted with permission from Ref. [126] (Copyright 2023, Elsevier).

A new wearable sensor system using aptamers has been developed to continuously monitor cortisol levels in sweat (Figure 9). An electrochemical sensor with a gold electrode, immobilized by a pseudoknot-assisted aptamer, is paired with a flexible microfluidic device for sweat collection. The aptamer’s conformational flexibility provides exceptional cortisol specificity, allowing for the ongoing tracking of temporal variations. The pseudoknot’s presence also significantly diminishes background interference, enhancing sensitivity. The sensor exhibits a broad linear dynamic range from 1 pM to 1 μM, encompassing the physiological spectrum. It also boasts an extraordinary LOD for cortisol in sweat at sub-picomolar levels (0.2 pM). Real-time on-body testing on human subjects has successfully shown the sensor’s ability to continuously track cortisol level changes for durations of up to 90 min, even under stress conditions [126].

Zhang et al. demonstrated an electrochemical corneal biosensor for detecting dopamine (DA) in tears to monitor myopia diopters [127]. The biosensor was fabricated using a vapor-phase polymerization-enhanced ball milling method to create electroactive nanoelectrodes from PEDOT functionalized with sulfur-doped graphene (PEDOT–graphene). Subsequently, the enzyme tyrosinase (Tyr) receptor was covalently immobilized onto the PEDOT–graphene nanosheets. A wearable corneal biosensor was constructed by the electrodeposition of the PEDOT–graphene–Tyr, contoured to the eye’s anatomical structure. The biosensor exhibited outstanding performance in detecting DA in both in vivo and in vitro tests, showing high stability and selectivity. It achieved a sensitivity of 12.9 µA × 10^−3^ M^−1^ cm^−2^ and a detection limit of 101 × 10^−9^ M. Furthermore, tear samples from patients with defocus-induced myopia were analyzed using the biosensor, which demonstrated good sensitivity to myopia diopters, indicating a correlation between myopia diopters and DA levels in tears. The findings suggest that this corneal biosensor could be a promising tool for the early, real-time detection and prevention of myopia.

A novel, wearable, and cost-effective microfluidic electrochemical impedimetric immunosensor was developed using a 3D electrode network of MXene (Ti_3_C_2_T_x_)-loaded laser-burned graphene (LBG) flakes to non-invasively monitor cortisol in human sweat. PDMS served as the substrate for a flexible and stretchable patch sensor. The LBG was transferred onto the PDMS, but the transfer process caused disconnections among the LBG flakes, adversely affecting the electrochemical performance of the electrode. To address this, highly conductive MXene was introduced onto the electrode. The patch sensor, attached to the skin, collected sweat and channeled it to the chamber by natural pressure. The MXene/LBG/PDMS-based sensor demonstrated a detection limit and linearity range for cortisol of 88 pM and 0.01–100 nM, respectively [128].

The electrochemical sensor strip, tailored for low-volume point-of-care immunosensing and exemplified with prostate-specific antigen, achieves remarkable efficiency by requiring merely about 2 μL of sample volume. This reduction in sample volume is enabled by a nature-inspired wettability patterning technique using a PDMS-TiO_2_ nanocomposite coating, which creates distinct super-hydrophilic and super-hydrophobic areas on the electrode surface. Such surface modification allows for highly sensitive antigen detection with minimal sample volumes. The sensor strip offers a linear detection range from 0.0001 to 100 ng mL^−1^ and a detection limit of 0.0001 ng mL^−1^. Furthermore, the sensor’s performance is stable at various inclinations of the strip, confirming its suitability for wearable sensing applications. Additionally, the PDMS-TiO_2_ coating improves the electrochemical performance of the sensor by increasing the surface area without causing electrical interference [129]. The analytical performance of recently reported WEBSs for other biomarkers detection is summarized in Table 4.

## 4. Conclusions and Future Perspectives

Biosensing technology holds considerable potential for advancement and is employed in numerous biological domains. It significantly aids in the detection of diverse biomolecules, often possessing superior specificity, high sensitivity, and mobility. Wearable and flexible biosensors have several significant applications in the field of health and medical care, facilitating convenient real-time diagnosis and ongoing health monitoring. This is especially crucial for monitoring various health hazards that people encounter daily.

This review examines the latest developments in flexible and wearable biosensors based on hydrogels. These biosensors are made of CPs, making them a viable option for future wearable sensor fabrication. These sensors can be tuned to achieve desired characteristics using various techniques, ranging from molecular design to microstructural configuration. However, several obstacles and limitations must be overcome to advance their use and commercialization.

The unresolved challenges and some future issues are as follows. First, the conductivity of the hydrogel should be improved by optimizing various polymerization processes. It is necessary to enhance CP HGs’ physicochemical characteristics, self-healing properties, adhesiveness, biocompatibility, stability, and purity. Second, by combining different metal and/or inorganic nanocomposites with the hydrogel matrix, the ideal concentration of CP nanocomposite hydrogels should be determined, which is a significant obstacle is the biosensors’ sensitivity and repeatability. Therefore, future studies should focus on resolving these issues and improving the use of CP composite hydrogel-based biosensors. Additionally, further investigation and attention must be paid to the stability of nanomaterials under extended use. Moreover, to enhance the functionality of flexible and wearable biosensors, more research is required to determine the interactions and synergistic effects of various nanocomposite materials and CPs. Inorganic CPs and nanomaterials with better characteristics should be developed and produced for wearable and flexible hydrogel biosensors in the future for effective sensing. The creation of a hydrogel biosensor enables multiparameter detection and real-time monitoring for more precise and timely monitoring of several patient indicators. These biosensors can be integrated with artificial intelligence and computer science, which have gained popularity recently, to apply nano-biosensors and make the new biosensors more intelligent and practical. Introducing a binary solvent such as glycerin or constructing a hydrophobic organogel for CP HGs are efficient ways to improve the dehydration feature in the open air, offering CP HGs long-term sensing stability in extreme environments such as high temperature or cold environments. We believe that conducting hydrogel-based wearable and flexible biosensor research and development will raise the bar for performance and expand the range of applications for wearable biosensor technology.

## Figures and Tables

**Table 1:** Most-used CPs for WSs and their properties.

CPs	Advantages	Disadvantages
PPy	High conductivity	Brittle
	Ease of synthesis	Poor solubility
	Biocompatibility	Relatively expensive
	Stable in an oxidized form	Poor cycling stability
		Non-transparent
PANI	Low cost	Relatively poor conductivity
	Large specific surface area	Lack of flexibility
	High stability	Non-biodegradable
	Ease of synthesis	Low processability
PEDOT	Electrochemically stable	Poor solubility
	Tunable conductivity	Limited flexibility
	Environmentally stable	Hard to process
	Biocompatibility	

**Table 2:** Comparison of analytical characteristics for several WEBSs for glucose monitoring.

Composites	Linear Range (mM)	LOD (µM)	Sensitivity (μA mM^−1^ cm^−2^)	Merits	Demerits	Ref.
Pt/MXene CH *	0−8	29.15	3.43	Enhanced the stability Long-term monitoring	Sweat accumulation affects the patch	[106]
Ni-Co MOF/Ag/rGO/PU fiber	0.01−0.66	3.28	425.9	Good mechanical flexibility	Short linear range	[107]
Ni-Co MOF/Au/PDMS	0.02−0.79	4.25	205.1	Good mechanical flexibility Long-term monitoring	Short linear range	[108]
GOx/N-GQDs/PANI	0.05−0.5	34	28.2	Good mechanical flexibility Long-term monitoring	Short linear range	[109]
PB-PEDOT NC	0.00625−0.8	4.0	-	Natural sweating sample	Hydrogel patch used only for sweat collection Non-flexible sensor	[110]
HA/MA-rGO-PANI	0.0003–0.005	0.3	421.42	Good mechanical flexibility	Short linear range	[111]
PET/QCS-MOx-OD/GOx	1.0–111	32.4	176	Self-healing ability	Hydrogel removal on PET surface by simple press	[112]
GOD/PB/RGO/SF	0.07–2.0 2.0–6.0 6.0–10	65.57	230.96 39.4 14.89	Chitosan hydrogel provides biocompatibility for enzyme	Poor long-term stability	[113]
PVA/CA/β-CD/GOx	0–0.5	98.84	8.762 V/mM	Triboelectric biosensors Self-healing ability Self-powered	Short linear range	[114]
PEDOT:PSS/DF/PB/GOx	0.001–0.243 0.243–3.243	0.85	340.1184.3	In vivo non-invasive monitoring of interstitial fluid glucose Reverse iontophoresis	Poor long-term storage stability	[115]
Ni-Co MOF/CNTs/MWCNTs/PDMS	0.02–1.1	6.78	71.62	Stamping-vacuum filtration dry transfer method Good mechanical flexibility	External sweat absorbent cloth	[116]

* Pt/MXene CH: platinum/MXene conductive hydrogel; Ni-Co MOF/Ag/rGO/PU fiber: Ni-Co metal–organic framework/Ag/reduced graphene oxide/polyurethane; Ni-Co MOF/Au/PDMS: Ni-Co metal–organic framework nanosheet-coated Au/polydimethylsiloxane; GOx/N-GQDs/PANI: glucose oxidase/N-doped graphene quantum dots; PB-PEDOT NC: Prussian Blue-doped poly(3,4-ethylenedioxythiophene nanocomposite; HA/MA-rGO-PANI: hyaluronic acid/methacrylic anhydride-reduced graphene oxide–polyaniline; PET/QCS-MOx-OD/GOx: polyethylene terephthalate/quaternized chitosan–CeO_2_/MnO_2_ nanospheres–oxidized dextran/glucose oxidase; GOD/PB/RGO/SF: glucose oxidase/Prussian Blue/reduced graphene oxide/silk fibroin; PVA/CA/β-CD/GOx: polyvinyl alcohol/citric acid/β-cyclodextrin/glucose oxidase; PEDOT:PSS/DF/PB/GOx: poly(3,4-ethylenedioxythiophene/dimethyl sulfoxide and Zonyl FS-300/Prussian blue/glucose oxidase; Ni-Co MOF/CNTs/MWCNTs/PDMS: Ni-Co metal–organic framework/carbon nanotubes/multi-walled carbon nanotubes/polydimethylsiloxane.

**Table 3:** Comparison of analytical characteristics for several WEBSs for lactate detection.

Composites	Linear Range (mM)	LOD (µM)	Sensitivity (μA mM^−1^ cm^−2^)	Merits	Demerits	Ref.
PC-AuNPs-LOx-CS *				Simultaneously sweat lactate and temperature detection	External sweat absorbent pad	[120]
	0.01−35	0.144	0.0824 μA mM^–1^			
		
LOx/PCP	0.02–1.0	0.75	-	Real-time monitoring of lactate released from C6 glioma cells	Short linear range	[121]
Alg/PEDOT/GNP/LOx-h				Good mechanical flexibility	Poor reproducibility	[122]
	1.0−100	400	0.0216			
		
		
PVA/CNCs@PDA-AuNPs/LOx	0.5−30	310	0.098 μA mM^–1^	Good mechanical flexibility Self-healing ability	High LOD	[123]
C/PB/GR/LOx	0.0−15	0.35	9.0	Zero-power sweat sampling patch Osmotic sweat extraction	Long-term stability not reported	[124]

* PC-AuNPs-LOx-CS: nanoporous polycarbonate–gold nanoparticles–lactate oxidase–chitosan; LOx/PCP: lactate oxidase/PB NPs/CNTs/PEDOT; Alg/PEDOT/GNP/LOx-h: alginate/poly(3,4-ethylenedioxythiophene)/gold nanoparticles/lactate oxidase–hydrogel; PVA/CNCs@PDA-AuNPs/LOx: poly(vinyl alcohol)/cellulose nanocrystals@polydopamine–gold nanoparticles/lactate oxidase; C/PB/GR/LOx: carbon ink/Prussian Blue/graphene/lactate oxidase.

**Table 4:** Comparison of analytical characteristics for several WEBSs for other biomarker detection.

Composites	Biomarker	Linear Range	LOD	Sensitivity	Merits	Demerits	Ref.
TA-Ag-CNT-PANI *	Tyrosine	0.01−0.2 mM	3.3 µM	-	Good mechanical elasticity Antibacterial property	Hydrogel lifetime is limited by water uptake	[125]
PDMS/Gold/Actin/2-ME	Cortisol	1.0 pM–1.0 µM	0.2 pM	0.33 μA/mm^2^/pM	Pseudoknot-assisted aptamer	Expensive than other methods	[126]
PEDOT-G-TYR	Dopamine (DA)	0.0–70 µM	101 nM	12.9 μA mM^−1^ cm^−2^	DA in tears was linked to myopia Biocompatibility	80% of the DA was lost from the eye during the in vivo test	[127]
Ti_3_C_2_T_x_ MXene/LBG	Cortisol	0.01−100 nM	3.88 pM	-	Low-cost immunosensor Non-invasive Rapid microfluidic analysis	Short linear range	[128]
PDMS-TiO_2_/PET	Model biomarker (Prostate-specific antigen)	0.0001−100 ng mL^−1^	0.0001 ng mL^−1^	30.3 µA ng^−1^ mL	Low-volume (2 μL) point-of-care immunosensor	In vivo not studied	[129]

* TA-Ag-CNT-PANI: tannic acid–Ag–carbon nanotube–polyaniline; PDMS/Gold/Actin/2-ME: polydimethylsiloxane/gold/actin/2-mercaptoethanol; PEDOT-G-TYR: poly(3,4-ethylenedioxythiophene)-functionalized sulfur-doped graphene–tyrosinase; Ti_3_C_2_T_x_ MXene/LBG: Ti_3_C_2_T*_x_* MXene/laser-burned graphene; PDMS-TiO_2_/PET: polydimethylsiloxane-titanium dioxide nanocomposite-coated carbon screen printed on polyethylene terephthalate.

## Data Availability

Not applicable.
